# The complete chloroplast genome sequence of *Hibiscus coccineus*

**DOI:** 10.1080/23802359.2021.2022545

**Published:** 2022-01-19

**Authors:** Zhiquan Wang, Hong Yang, Fengjiao Zhang, Yunlong Yin, Chunsun Gu

**Affiliations:** a Institute of Botany, Jiangsu Province and Chinese Academy of Sciences, Nanjing, China; b Jiangsu Provincial Platform for Conservation and Utilization of Agricultural Germplasm, Jiangsu Key Laboratory for the Research and Utilization of Plant Resources, Nanjing, China; c College of Forest Sciences, Nanjing Forestry University, Nanjing, China

**Keywords:** *Hibiscus coccineus*, complete chloroplast genome, phylogenetic analysis

## Abstract

*Hibiscus coccineus* is famous for its wide geographical distribution and the showy flowers of scarlet rose mallow. It belongs to the Malvaceae family and has greatly ornamental and ecological value. In this study, high-throughput sequencing and bioinformatics technology were used to assemble the complete chloroplast genome sequence, which will provide more genomic information for studying the genetic diversity and phylogenetic relationship. The full length of chloroplast genome is 160,280 bp, composed of a large single-copy (LSC) region of 89,121 bp, a small single-copy (SSC) region of 18,673 bp, and two inverted repeats (IRs) of 26,243 bp. A total of 113 genes were annotated, including 79 protein-coding genes, 30 tRNA, and four rRNA genes. Phylogenetic tree analysis revealed that the *Hibiscus coccineus* is closest to *Hibiscus mutabilis* in the *Hibiscus* L.

*Hibiscus* L. is a genus within the Malvaceae family, which comprises about 300 species mainly distributed in tropical and subtropical regions (Bruna et al. 2009; Shaheen et al. 2009). *Hibiscus coccineus*_Walter 1788 (*H. coccineus*) is one of species within the *Hibiscus* section Muenchhusia (Small 2004; Kuligowska et al. 2016). The perennial nature, native status, habitat, and showy flowers of scarlet rosemallow have made this species a popular garden plant and classified as an obligate wetland plant (Gettys 2012). Some DNA sequencing methods were performed to clarify the phylogenetic analysis in the genus (Pfeil et al. 2002; Small 2004; Sundar et al. 2016; Werner et al. 2016), but complete chloroplast genome is rarely reported. In this work, we obtained the complete sequence of the chloroplast genome of *H. coccineus* using next-generation sequencing technology and analyzed its phylogenetic relationship, which would be helpful for further study on the identification and classification of *Hibiscus* L.

Total genomic DNA of *H. coccineus* was collected from Nanjing Botanical Garden, Mem. Sun Yat-sen (118°49′55″E, 32°3′32″N), Nanjing, China. The specimen and DNA were deposited in Nanjing Botanical Garden, Mem. Sun Yat-sen (Zhiquan Wang, wangzhiquan@cnbg.net) under the voucher number NBG-HC-0001. Total DNA was extracted using the CTAB method. A paired-end library with an insert-size of 350 bp was constructed and sequenced on the Illumina NovaSeq 6000 system (Illumina, San Diego, CA). In total, ∼8 Gb of clean data (24,754,074 reads) were obtained. The complete chloroplast genome was assembled using GETORGANELLE pipeline (Jin et al. 2020), and annotated using Geneious Prime v.2021.1.1 (http://www.geneious.com) taking *H. cannabinus* (NC045873) as a reference. The annotated chloroplast genome sequence of *H. coccineus* was deposited in GenBank (no. OK336487).

The whole chloroplast genome sequence of *H. coccineus* was 160,280 bp in length, including a large single-copy (LSC) region of 89,121 bp and a small single-copy (SSC) region of 18,673 bp separated by two inverted repeats (IRs, including IRa and IRb) of 26,243 bp. The *H. coccineus* chloroplast genome contained a total of 113 genes, including 79 protein-coding genes, 30 transfer RNA genes, and four ribosomal RNA genes. Seven protein coding genes, four rRNA genes and seven tRNA genes are duplicated in the IR regions. There are 17 chloroplast genes harbored introns, among which 15 genes contained single introns, and two genes (*ycf3*, *clpP*) contained two introns. The overall GC content of the complete chloroplast genome was 36.9%.

To determine the phylogenetic status of *H. coccineus*, eight other chloroplast genome sequences were obtained from the GenBank database. The phylogenetic tree was reconstructed using the maximum-likelihood (ML) method based on the multiple alignment of *H. coccineus* and other seven previously reported chloroplast genomes of *Hibiscus*, with *Gossypium herbaceum* (JF317353) as an outgroup. ML analysis was conducted using PhyML v.3.0 (Guindon et al. 2010) with 1000 bootstrap replicates. In addition, the phylogenetic tree showed that *H. coccineus* clustered in the genus *Hibiscus* L., and was more closely related with *H. mutabilis* (Figure 1). The complete sequence of *H. coccineus* chloroplast genome will be helpful for further studies in molecular markers and molecular breeding.

**Figure 1.**
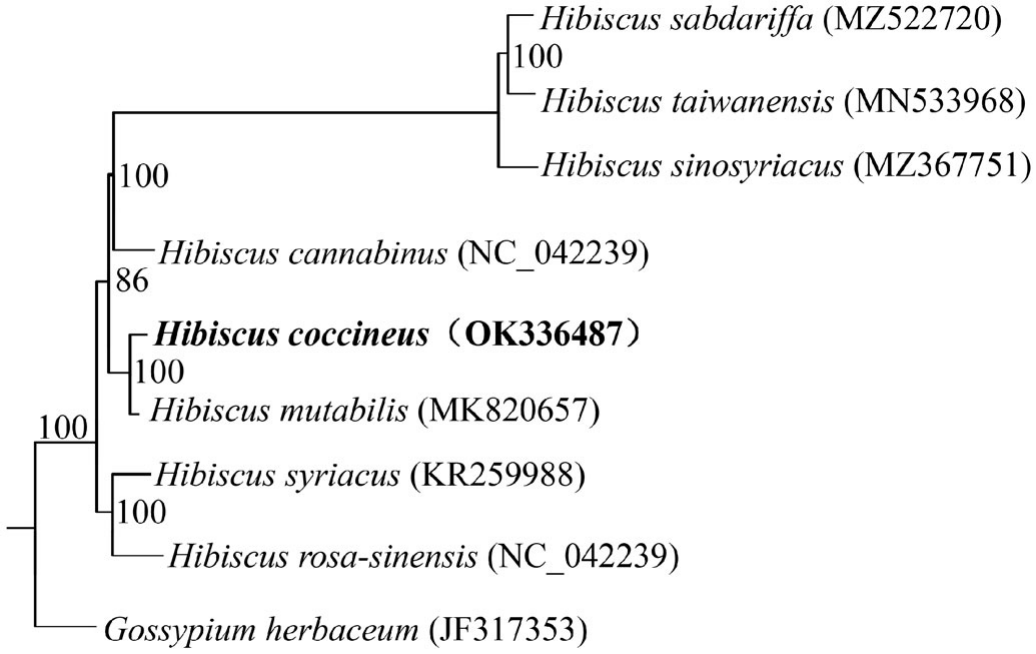
Phylogenetic tree based on the chloroplast genome sequences of nine species, showing the close relationship between *H. coccineus* and *H. mutabilis*. Numbers next to the nodes indicate the bootstrap value from 1000 replicates. GenBank accession no. of each species was shown in the brackets after names.

## Data Availability

The genome sequence data that support the findings of this study are openly available in GenBank of NCBI at https://www.ncbi.nlm.nih.gov under the accession no. OK336487. The associated BioProject, SRA, and Bio-Sample numbers are PRJNA767224, SRR16111513, and SAMN21894747, respectively.
